# Circulating Gastric Cancer Stem Cells as Blood Screening and Prognosis Factor in Gastric Cancer

**DOI:** 10.1155/2024/9999155

**Published:** 2024-08-08

**Authors:** Jared Becerril-Rico, Julian Grandvallet-Contreras, M. Patricia Ruíz-León, Sebastián Dorantes-Cano, Lizbeth Ramírez-Vidal, José M. Tinajero-Rodríguez, Elizabeth Ortiz-Sánchez

**Affiliations:** ^1^ Subdirección de Investigación Básica Instituto Nacional de Cancerología, Secretaría de Salud, Mexico City, Mexico; ^2^ Posgrado en Ciencias Biológicas Universidad Nacional Autónoma de México, Mexico City, Mexico; ^3^ Posgrado en Ciencias Biomédicas Universidad Nacional Autónoma de México Circuito Exterior s/n Ciudad Universitaria, Coyoacán, Mexico City 04510, Mexico; ^4^ Doctorado en Ciencias Biomédicas Facultad de Ciencias Químico Biológicas Universidad Autónoma de Guerrero, Av. Lázaro Cárdenas S/N, Ciudad Universitaria, Chilpancingo 39090, Guerrero, Mexico

## Abstract

Gastric cancer (GC) is the fourth leading cause of cancer-related death, associated with late diagnosis and treatment resistance. Currently, screening tests for GC are not cost-effective or have low accuracy. Previously, we described an extended phenotype of gastric cancer stem cells (GCSCs; CD24^+^CD44^+^CD54^+^EpCAM^+^) that is associated with metastasis and tumor stage in GC patients. The goal of the current research is to evaluate the presence of these GCSCs in the peripheral blood of GC patients and healthy volunteers. A total of 73 blood samples were collected from 32 GC patients and 41 healthy volunteers. After peripheral blood mononuclear cell (PBMC) extraction, multiparametric flow cytometry was performed looking for GCSCs. Using clustering data through artificial intelligence (AI), we defined high/low levels of circulating GCSCs (cGCSCs) and proceeded to evaluate its association with clinical and prognostic variables. Finally, a diagnostic test analysis was performed evaluating patients and healthy volunteers. We found that cGCSCs are present in most GC patients with a mean concentration of 0.48%. The AI clustering showed two groups with different cGCSC levels and clinical characteristics. Through statistical analysis, we confirmed the association between cGCSC levels and lymph node metastasis, distant metastasis, and overall survival. The diagnostic test analysis showed sensibility, specificity, and area under the curve (AUC) of 83%, 95%, and 0.911, respectively. Our results suggest that the assessment of cGCSCs CD24^+^CD44^+^CD54^+^EpCAM^+^ could be a potential noninvasive test, with prognostic value, as well as highly sensitive and specific for screening or diagnosis of GC; however, a larger scale study will be necessary to confirm this.

## 1. Introduction

Gastric cancer (GC) is the 5^th^ cancer type with the highest incidence worldwide as well as the 4^th^ cause of cancer-related death [1]. Mortality rate is related with GC stage at diagnosis, resistance to treatment, and disease relapse [2].

There are at least four main screening methods for GC: upper gastrointestinal series, serum pepsinogen, serum anti-*Helicobacter pylori* IgG assay, and upper endoscopy. Upper endoscopy has the highest rate for detecting GC; however, large-scale screening showed that this method is not cost-effective in low-incidence countries. Furthermore, it is an invasive and operator-dependent test [3].

Despite the several treatment options available for GC patients, recurrence is frequent [4]. In recent years, several models of tumor growth and behavior have tried to explain it. Among these, the cancer stem cell (CSC) model has stood out. This model describes subsets of cancer cells with different potential for proliferation and differentiation, where CSCs are the subset with the highest potential to proliferate and differentiate, as well as having self-renewal capacity, ability to initiate new tumors in xenograft models, high metastatic capability, and resistance to chemotherapy and radiotherapy [5]. Therefore, the existence of gastric cancer stem cells (GCSCs) can partly explain relapse, treatment resistance, and high incidence of metastasis in GC patients [6].

Many markers for easy identification of GCSCs have been studied, among which surface markers like CD24, EpCAM, CD49f, CD54, LGR5, and CD133 have been more effective, as well as transcription factors such as Nanog, Sox2, and Oct4. However, all those markers are also present in normal tissue, which complicates their use in GC diagnosis or screening and detection of therapeutic targets, as well as prognosis or staging factors [7]. In this sense, a multimarker phenotype of GCSCs may be a viable option to increase the specificity of GCSC identification, improving their clinical applicability.

Previously, we described an extended phenotype of GCSCs (CD24^+^CD44^+^CD54^+^EpCAM^+^) related with metastasis and cancer stage in 127 patients [8]. Here, we report the presence of cells with this extended phenotype in the peripherical blood of patients with GC and its potential use as a noninvasive screening test for GC.

## 2. Materials and Methods

### 2.1. Sample Collection

This study includes a total of 73 blood samples collected from 32 patients diagnosed with GC and 41 healthy volunteers. GC patients were recruited from the “Instituto Nacional de Cancerología” (Mexico City, Mexico) between February 2020 and December 2021, all of them with GC confirmed by biopsy, age ≥ 18 years old, no history or concurrence of any additional malignancy, nonrecurrent GC, no diagnosis of any autoimmune disease, and without prior antitumoral treatment; patients of any histologic type and tumor stage were included. Healthy volunteers were used as negative controls, recruiting subjects without GC diagnosis and without a diagnosis of autoimmune disease. All patients were taken under informed consent approved by the “Comité de Investigación” and the “Comité de Bioética en Investigación” (Research and Bioethics committees) from the institute (Registration numbers 015/011/OMI; CEI/934/15 and 015/011/IBI; CEI/934/15). All subjects were included in the associative statistical analysis and artificial intelligence methods; however, only the subjects with a follow-up time >4 months were included in the survival analysis to avoid bias in the results derived from not yet observable long-term outcomes in subjects with a follow-up time <4 months.

### 2.2. Blood Sample Preparation

For each enrolled subject, we collected 5–10 mL of blood in EDTA vacutainer tubes, and they were processed immediately. Extraction of peripheral blood mononuclear cells (PBMC) was performed using density gradient separation with Lymphoprep™ (Stem Cell Technologies, Oslo, Norway) according to the manufacturer's protocol. After PBMC recovery, cells were washed with 8-ml phosphate-buffered saline (PBS) and centrifuged at 400 *g* for 10 min. Finally, mononuclear cells were diluted in PBS containing 0.5% bovine serum albumin (BSA) and 2 mM EDTA for flow cytometry assays.

### 2.3. Flow Cytometry

Cell staining was performed with antibodies diluted 1 : 100 in 100 *μ*L of PBS containing 0.5% BSA and 2 mM EDTA. The antibodies used were anti-CD24 (PE, clone ML5, BioLegend), CD44 (FITC, clone BJ18, BioLegend), EpCAM (PE/Cyanine7, clone 9C4, BioLegend), CD54 (Pacific Blue, clone HA58, BioLegend), and CD45 (APC, clone HI30, BioLegend). After 25 min of incubation, cells were washed twice with PBS containing 0.5% BSA and finally fixed with 1% paraformaldehyde in PBS. Cell acquisition was performed with BD FACSAria™ II flow cytometer. FlowJo 10.4 software was used for analysis.

### 2.4. Statistical Analysis

Relational analysis between categorical groups was evaluated using *X*^2^ test. Kaplan–Meier graph and log-rank tests were used for survival analysis. We used the IBM SPSS Statistics version 28 for all statistical analysis, considering *p* < 0.05 as a significant critical value.

### 2.5. Artificial Intelligence Analysis

For data clustering with artificial intelligence, Jupyter Notebook for Python programming was used. The initial database with clinical and GCSCs data was preprocessed, converting categorical variables to dummy variables (only values of 1 and 0), and numerical data were standardized. Next, we used the MiniBatchKMeans algorithm from scikit-learn library, and data clustering prediction was performed. Clinical characteristics were summarized according to each clustering group. For the graphic demonstration of the data clustering, we reduced the dimensionality of clinical variables (10 variables) to only two singular vectors using the TruncatedSVD algorithm.

## 3. Results

### 3.1. Circulating GCSCs Are Associated with Metastasis and Survival

**Figure 1:**
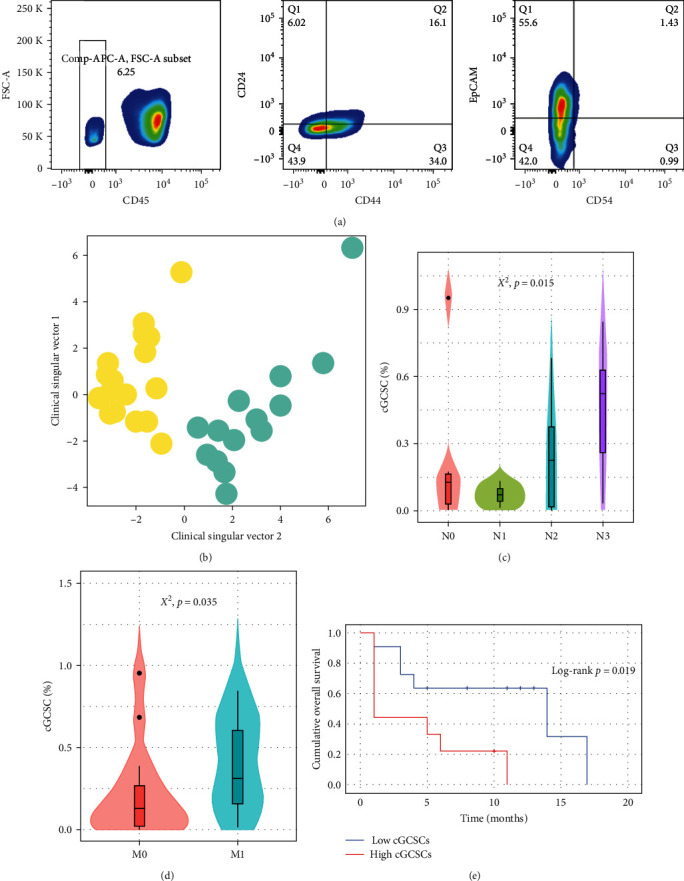
cGCSCs are related with clinical characteristics and prognosis in GC patients: (a) analysis strategy in flow cytometry to evaluate cGCSCs (CD24^+^CD44^+^CD54^+^EpCAM^+^); (b) reducing dimensionality of clinical variables with artificial intelligence, it is possible to observe the two patient groups predicted by the MiniBatchKMeans algorithm, where the low cGCSC group is shown in blue and the high cGCSC group is in yellow; (c and d) stage of invasion to lymph nodes and distant metastasis according to cGCSCs %; and (e) Kaplan–Meier survival plot by cGCSC level.

In order to propose a noninvasive and accessible diagnosis for GC, we considered the hematogenous migration of GCSCs with the phenotype CD24^+^CD44^+^EpCAM^+^CD54^+^ (hereafter mentioned as GCSCs), previously evaluated in zebra fish [8]. From this observation, we hypothesized that these GCSCs could be found in the peripheral blood of patients with GC. Then, we evaluated the peripheral blood from 32 GC patients searching for GCSCs for prior selection of CD45^−^ cells (Figure 1(a)). Patients included 21 men and 11 women, with a mean age of 58.03 years (±12.75), and follow-up was carried out for a mean time of 6.4 months (±5.18). The clinical characteristics of GC patients included are described in Table 1.

**Figure 2:**
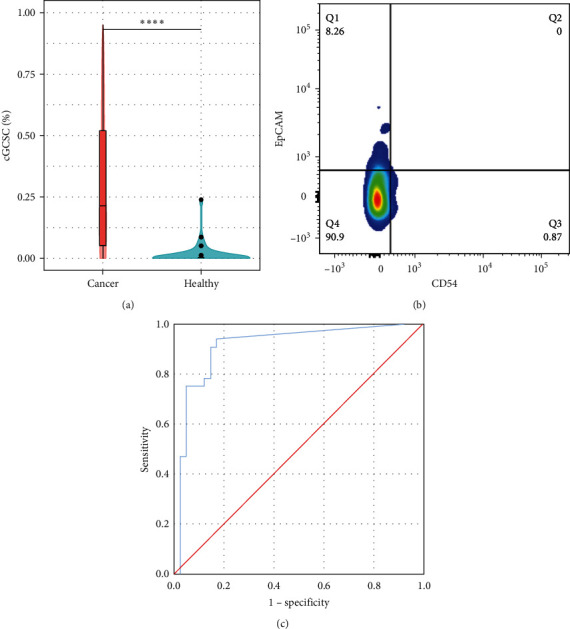
cGCSCs in healthy volunteers and their potential as diagnostic test: (a) percentage of cGCSCs in GC patients and healthy volunteers, (b) flow cytometry analysis in healthy volunteer (#17), where CD24^+^CD44^+^ circulating cells have a EpCAM+ population but not a double-positive population, and (c) ROC curve graph with 0% of cGCSCs as cutoff for positive/negative test.  ^*∗∗∗∗*^*p* < 0.0001.

Circulating GCSCs (cGCSCs) were detected in 30 of 32 GC patients (93.75%) with a mean cell percentage of 0.48% (±0.65; Figure 2(a)).

To assess whether cGCSC concentration was different according to clinical characteristics, we initially used the artificial intelligence algorithm MiniBatchKMeans to find possible patient groups through data clustering [9]. This strategy allowed us to find patient cohorts based on all the clinical characteristics of the subjects and not based on a limited number of variables as performed by classical statistical clustering tools. The algorithm found two patient groups (Figure 1(b)) with differences in cGCSC percentage and clinical characteristics (Table 2). According to the analysis based on artificial intelligence, the group with higher cGCSCs (yellow in Figure 1(b)) was distinguished by advanced Tumor Nodes Metastases (TNM) stage, higher T stage, lower survival, diffuse histologic type, and middle-aged adults, as well as M1 stage with distant metastasis mainly to the liver. Conversely, the group with low cGCSCs (blue) had undefined tendencies in most of its clinical characteristics, although it exhibited characteristics related to nonmetastatic cancer (Figures 1(c) and 1(d)), as well as greater mean survival (Figure 1(e)).

Based on the clustering generated using machine learning, cGCSCs were categorized as high/low level (0.30% as cutoff). Then, we performed a *X*^2^ test to statistically confirm the association between cGCSC levels and clinical characteristics (Table 2). We observed that high cGCSC level shows a significant association with lymph node and distant metastasis (Figures 1(c) and 1(d)). In addition, survival analysis was performed, and using a Kaplan–Meier survival plot, we found that high levels of cGCSCs are related to lower survival in GC patients (Figure 1(e)). Therefore, GCSCs with the phenotype CD24^+^CD44^+^CD54^+^EpCAM^+^ can be found in the peripheral blood of GC patients, and the high level of these cells is related with local and distant metastasis, as well as survival.

### 3.2. cGCSCs Are Present in GC Patients, but Not in Healthy Volunteers

To know whether cGCSCs are specifically found in GC patients, we evaluated the peripheral blood of 41 healthy volunteers, 22 women and 19 men, with a mean age of 34.4 years (±15.13). cGCSCs were in 6 of 41 healthy volunteers (14.63%), with a cell concentration of 0.091% (±0.519), which shows a significant difference with GC patients (*p* < 0.0001; Figure 2(a)). There were no differences in sex distribution between GC patients and healthy volunteers (*p*=0.150). Healthy volunteers were significantly younger than GC patients (*p*=0.001), but age was not associated with cGCSC percentage (*p*=0.114); therefore, the clinical value of cGCSCs shown in this manuscript is not influenced by the age difference between cohorts.

In addition, analysis of cell phenotype in the peripheral blood from healthy volunteers showed the presence of cells with the phenotype CD24^+^CD44^+^EpCAM^+^CD54^−^ but rarely CD24^+^CD44^+^CD54^+^EpCAM^+^, which establishes a difference in circulating cell phenotypes (Figure 2(b)). In support of this, there are reports that propose CD54 as a marker related with CSCs and metastasis [10].

### 3.3. Evaluation of cGCSCs Could be Used as a Potential Diagnostic Test in GC

Considering GC patients and volunteers who were positive or negative for the presence of cGCSCs, we can structure a contingency table of diagnostic tests (Table 3).

Sensitivity and specificity for our cGCSC test were 83% and 95%, while in receiver operator characteristic (ROC) curve analysis, the area under the curve (AUC) obtained was 0.911 (*p* < 0.0001; Figure 2(c)). These results determine a diagnosis accuracy of 90.27%, which allows us to propose the cGCSC (CD24^+^CD44^+^CD54^+^EpCAM^+^) evaluation as a potential noninvasive diagnostic or screening test for GC.

## 4. Discussion

In the last couple of decades, there have been advances in the detection of circulating GC cells using a diagnostic approach; however, the commonly used methodologies are PCR or magnetic isolation with a reported sensitivity of 35% and 82%, respectively [11]. In this study, we showed the presence of GCSCs with the CD24^+^CD44^+^CD54^+^EpCAM^+^ phenotype in the peripheral blood of GC patients using flow cytometry. Furthermore, the flow cytometry tool has several methodological advantages such as precise cell identification, semiquantitativeor quantitative determination, fast sample evaluation, wide equipment availability, and low cost per test [12]. These advantages make it possible to propose the evaluation of circulating cells through flow cytometry as a feasible screening test for GC.

The extended phenotype of cGCSCs used in the present study provides high specificity for the identification of GC patients. Furthermore, the metastatic potential of these cGCSCs allows their easy detection in peripheral blood. There are a few investigations that have reported GCSCs in peripheral blood, for example, Chen et al. demonstrated that CD44^+^CD54^+^ GCSCs can be found in peripheral blood from GC patients and after xenotransplatation are able to generate tumors in mice. However, the authors do not provide statistical results about those evaluations [13]. Similarly, Watanabe et al. [14] described circulating cells with a EpCAM^+^CD44^+^ phenotype in GC patients, reporting a sensitivity of 92.3%, specificity of 100%, and AUC of 0.974, although specificity and AUC could be questionable because they enrolled only 12 healthy volunteers compared with 26 patients. One of the major limitations of our study was the low number of patients included, since we include a total of 73 patients, 32 GC patients, and 41 healthy volunteers. However, it provides an initial approach necessary to support a larger scale diagnostic Phase II study. Additionally, other similar studies have enrolled 35–38 total participants [13, 14, 15, 16]; therefore, our study is similar or more appropriate in patient number.

Additionally, circulating CD44^+^ cells have been associated with local and distant metastasis, and circulating CD133^+^ cells are related with cell differentiation, local metastasis, and survival, but none of these investigations evaluated the potential for diagnosis [15, 16]. Thus, the association between circulating cells and clinical variables provides an additional advantage, since a noninvasive test, similar to the one proposed here, may be sufficient for screening and staging of the disease, allowing faster decisions in clinical management and improving the outcome for GC patients.

As mentioned before, we obtained high accuracy for GC detection, but comparing our results with other screening tests for GC, a meta-analysis reported that the serum pepsinogen test has a sensibility, specificity, and AUC for GC diagnosis of 69%, 73%, and 0.76, respectively [17]. Moreover, a Korean study with more than 2.5 million patients described that the sensitivity and specificity of upper gastrointestinal series was 36.7% and 69%, while upper endoscopy had 96.1% and 96% [18].

Research studies of diagnostic tests are usually divided into four stages: Phase I determines the normal results of the test in healthy volunteers; Phase IIa compares test results between diagnosed diseased participants and healthy participants; Phase IIb evaluates whether the test result is related with disease severity; Phase IIc examines the predictive value of the test in participants with suspected disease; Phase III determines the clinical effects of using the test result to guide clinical treatment; and Phase IV estimates long-term effects derived from introducing the new diagnostic test [19]. As described, the present study evaluated Phase I–IIb items, but a Phase II study with a large number of participants can elucidate the real diagnostic value of cGCSCs. Finally, our results seem promising in the sense of a quantitative method of screening with similar accuracy as upper endoscopy but through a noninvasive, faster, and cheaper method.

## 5. Conclusions

In summary, our results demonstrate that it's possible to find GCSCs with a CD24^+^CD44^+^CD54^+^EpCAM^+^ phenotype in the peripheral blood of most GC patients, and the level of these cGCSCs is related with local and distant metastasis, as well as overall survival. Our findings further indicate that this cell phenotype is absent or present at very low levels in healthy volunteers; therefore, performing a quantitative diagnostic test analysis, we observe high sensibility, specificity, and AUC, similar to conventional GC diagnostic tests. Therefore, the evaluation of cGCSCs in the peripheral blood of GC patients could be a potential noninvasive, fast, and cheap screening or diagnostic method; however, a larger scale study is needed to confirm it.

## Figures and Tables

**Table 1:** Clinical characteristics in patients with GC.

Clinical characteristic	*N* = 32	Percentage (%)
Sex		
Male	21	65.6
Female	11	34.4
Age group		
Young adults	3	9.4
Middle-aged adult	14	43.8
Old adults	15	46.9
Lauren classification		
Intestinal	10	31.3
Diffuse	18	56.3
Mixed	3	9.4
Tumor grade		
Grade I	1	3.1
Grade II	3	9.4
Grade III	28	87.5
cTNM classification		
I	1	3.1
IIA	2	6.3
IIB	1	3.1
IIIA	9	28.1
IIIB	0	0
IIIC	0	0
IV	19	59.4
T stage		
T1	1	3.1
T2	1	3.1
T3	3	9.4
T4a	10	31.3
T4b	17	53.1
N stage		
N0	6	18.8
N1	4	12.5
N2	9	28.1
N3	13	40.6
M stage		
M0	17	53.1
M1	15	46.9
Distant metastasis site		
Liver	7	21.9
Lymph node	4	12.5
Lung	2	6.3
Ovary	2	6.3
Bladder	1	3.1
No distant metastasis	16	50
Borrmann classification		
I	2	6.3
II	0	0
III	13	40.6
IV	13	40.6
V	4	12.5

**Table 2:** Statistical analysis and clustering according to cGCSC level.

Clinical characteristics	High cGCSCs	Low cGCSCs	*p* value (*X*^2^)
Mean cGCSC %	0.92%	0.09%	—
Mean survival	3.3 months	9.5 months	—
Sex	Undefined	Undefined	0.108
Age group	Middle-aged adult	Undefined	0.198
Lauren classification	Diffuse	Undefined	0.068
Tumor grade	Undefined	Undefined	0.545
cTNM stage	IV	Undefined	0.399
T stage	T4b	Undefined	0.685
N stage	Undefined	Undefined	**0.015**
M stage	M1	M0	**0.035**
Distant metastasis site	Liver	No distant metastasis	0.098
Borrmann classification	Undefined	Undefined	0.409

Bold numbers indicate statistically significant differences between high and low cGCSCs groups.

**Table 3:** Contingency table of diagnostic tests.

	cGCSCs test result		
Gastric cancer diagnosis	Positive	Negative	Total
Positive	30	2	32
Negative	6	35	41
Total	36	37	—

## Data Availability

The artificial intelligence algorithm and database used for data clustering are available in Zenodo repository, https://doi.org/10.5281/zenodo.6800478. All other data analyzed in the present study are included in this published article.

## Abbreviations

AUC: Area under the curve
BSA: Bovine serum albumin
CSCs: Cancer stem cells
cGCSCs: Circulating gastric cancer stem cells
GC: Gastric cancer
GCSCs: Gastric cancer stem cells
PBMC: Peripheral blood mononuclear cells
PBS: Phosphate buffered saline
ROC: Receiver operator characteristic.
